# Improvement in B1+-homogeneity of 3T cardiac MRI in swine with dual-source parallel RF excitation

**DOI:** 10.1186/1532-429X-15-S1-W22

**Published:** 2013-01-30

**Authors:** D Herzka, H Ding, M Schär

**Affiliations:** 1 Biomedical Engineering, Johns Hopkins University School of Medicine, Baltimore, MD, USA; 2 Biomedical Engineering, Tsinghua University, Beijing, China; 3 Philips Healthcare, Cleveland, OH, USA

## Background

Conventional MRI scanners up to a magnetic field strength of 3T use an integrated birdcage quadrature coil to generate a radio frequency (RF) excitation field (B1+). At 3T, Sung et al. observed a flip angle variation ranging from 31 to 66% over the entire left ventricle (LV) in humans as well as a flip-angle distribution from 34° to 63, for a nominal flip angle of 60°. This not only demonstrates that the B1+ field over the LV is inhomogeneous but also that the average flip angle (RF power setting) can be 20% lower than desired. Such erroneous flip angles may lead to local signal reduction, artifacts, failure of magnetization preparation pulses, and eventually to biased quantitative measures. Recently, it has been shown that multi-channel transmit systems can be used to reduce these inhomogeneities in humans by the use of RF-shimming. Here, we quantify improvements in B1+-homogeneity at 3T when using dual-source parallel RF excitation, and correlate results with animal size.

## Methods

Swine (N=22) were imaged repeatedly and at different times as part of other imaging studies. Animal weight varied between ~25 and 125 kg. A 3.0T MR system (Achieva TX, Philips Healthcare, Best, The Netherlands) and a 32-channel cardiac array were used. B1+-mapping was carried out before and after RF-shimming using a cardiac-triggered, breath-hold, saturated dual-angle method [6]. Data were acquired axially, during breath-hold, in diastole. A volume manually drawn over the heart, primarily through the ventricles was used to localize the shimming. N=44 independent imaging sessions were analyzed. An elliptical ROI was drawn using the magnitude image as a guide, aiming to cover the whole heart. Pixel values where extracted from the B1+-map and represent the percent of the desired flip angle achieved. Animal size was characterized by drawing an additional elliptical ROI encompassing the whole animal.

## Results

**Figure 1:**
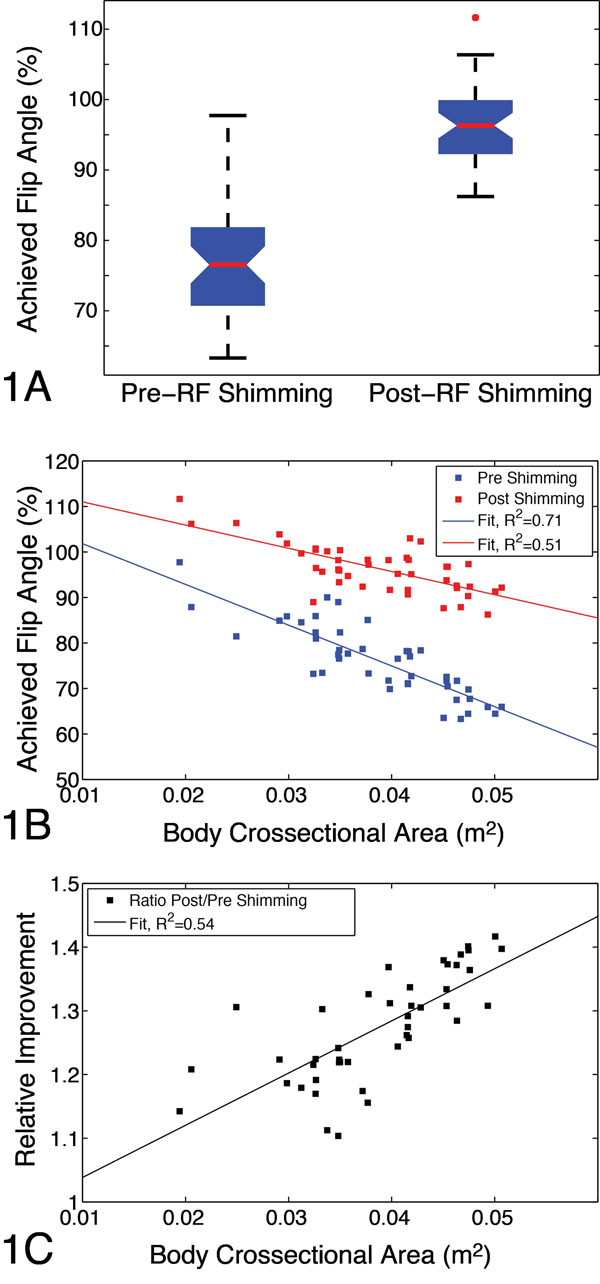
A) Box plot of percentage of the desired flip angle achieved in the cardiac ROI before and after RF-shimming. Correlation of cross-sectional area with achieved flip angle (%) (B) and with ratio of post-to-pre RF-shimming improvement in achieved flip angle (C). Significant correlations are observed indicating that animal size reduces achieved flip angle (as expected), but also that RF shimming has a more positive effect when imaging larger animals.

In all cases the average percent flip angle achieved increased after RF-shimming. Without B1+-shimming, cardiac ROIs had a mean value of 76.1%±8.0 vs. 96.4%±5.4 with B1+-shimming (p<<1e-10, Fig 1A). The cross-sectional area of each animal was correlated to both pre-and post achieved flip angle (R2=0.71, 0.51, respectively) but less so for values measured after RF-shimming (Fig 1B). The relative improvement, ratio of post-to-pre achieved flip angle percentage, was also positively correlated to cross-sectional area (R2=0.54, Fig 1C).

## Conclusions

The use of localized RF shimming with dual sources significantly increases the effective flip angle. These improvements should have significant effects in SNR and the predictability of image quality since current cardiac imaging involves high flip angle RF pulses whose performance can be affected by B1+ heterogeneity. Here we show that not only does the degree of loss due to dielectric effects depend on animal size, so does the magnitude of the improvement in B1+ homogeneity.

## Funding

This work was funded in part by AHA-11SDG5280025.

